# The Prognostic and Predictive Role of Epidermal Growth Factor Receptor in Surgical Resected Pancreatic Cancer

**DOI:** 10.3390/ijms17071090

**Published:** 2016-07-08

**Authors:** Meng Guo, Guopei Luo, Chen Liu, He Cheng, Yu Lu, Kaizhou Jin, Zuqiang Liu, Jiang Long, Liang Liu, Jin Xu, Dan Huang, Quanxing Ni, Xianjun Yu

**Affiliations:** 1 Pancreatic Cancer Institute, Fudan University, Shanghai 200032, China; guomeng@fudanpci.org (M.G.); liuchen@fudanpci.org (C.L.); chenghe@fudanpci.org (H.C.); luyu@fudanpci.org (Y.L.); jinkaizhou@fudanpci.org (K.J.); liuzuqiang@fudanpci.org (Z.L.); longjiang@fudanpci.org (J.L.); liuliang@fudanpci.org (L.L.); xujin@fudanpci.org (J.X.); niquanxing@fudanpci.org (Q.N.); 2 Department of Pancreas Surgery, Fudan University Shanghai Cancer Center, Shanghai 200032, China; 3 Department of Oncology, Shanghai Medical College, Fudan University, Shanghai 200032, China; ilytty@163.com; 4 Department of Pathology, Fudan University Shanghai Cancer Center, Shanghai 200032, China

**Keywords:** pancreatic ductal adenocarcinoma, EGFR status, overall survival, adjuvant therapy, prognosis

## Abstract

The data regarding the prognostic significance of EGFR (epidermal growth factor receptor) expression and adjuvant therapy in patients with resected pancreatic cancer are insufficient. We retrospectively investigated EGFR status in 357 resected PDAC (pancreatic duct adenocarcinoma) patients using tissue immunohistochemistry and validated the possible role of EGFR expression in predicting prognosis. The analysis was based on excluding the multiple confounding parameters. A negative association was found between overall EGFR status and postoperative survival (*p* = 0.986). Remarkably, adjuvant chemotherapy and radiotherapy were significantly associated with favorable postoperative survival, which prolonged median overall survival (OS) for 5.8 and 10.2 months (*p* = 0.009 and *p* = 0.006, respectively). Kaplan–Meier analysis showed that adjuvant chemotherapy correlated with an obvious survival benefit in the EGFR-positive subgroup rather than in the EGFR-negative subgroup. In the subgroup analyses, chemotherapy was highly associated with increased postoperative survival in the EGFR-positive subgroup (*p* = 0.002), and radiotherapy had a significant survival benefit in the EGFR-negative subgroup (*p* = 0.029). This study demonstrated that EGFR expression is not correlated with outcome in resected pancreatic cancer patients. Adjuvant chemotherapy and radiotherapy were significantly associated with improved survival in contrary EGFR expressing subgroup. Further studies of EGFR as a potential target for pancreatic cancer treatment are warranted.

## 1. Introduction

Pancreatic ductal adenocarcinoma (PDAC) is the fourth leading cause of death for all devastating human malignancies [1,2,3]. Surgical resection is still the only potentially curative therapeutic option [3]. Unfortunately, upon the diagnosis of PDAC, the majority of patients have an advanced stage of disease and cannot be cured by resection [4]. Even if a potentially curative resection can be performed, the median survival is shorter than 20 months, and the rate of five-year overall survival is less than 20% [2,4,5,6]. Due to the frequency of local recurrence and distant metastases after resection, adjuvant therapy is necessary to decrease the risk of locoregional recurrence and distant metastases [3,4,5,7]. However, the significance of different adjuvant strategies in pancreatic cancer are inconclusively and insufficiently reported in previous studies [8,9,10]. Multiple prognostic factors for PDAC (pancreatic duct adenocarcinoma) have been thoroughly studied and include gender, age, size and location of the tumor, tumor stage, lymph node metastasis status, tumor grade, and serum carbohydrate antigen 19-9 level, but the sensitivities of these prognostic factors are limited in the absence of carcinoma-associated proteins expression profiles [4].

Human epidermal growth factor receptor (EGFR (epidermal growth factor receptor) or HER1) is a member of the transmembrane tyrosine kinase receptor family and has been shown to be involved in the regulation of growth, differentiation and proliferation in solid tumors [11,12,13]. Overexpression of EGFR has been discovered in a wide variety of solid tumors, including breast, prostate, colon and non-small cell lung neoplasms [11,12,13,14]. For pancreatic adenocarcinoma, EGFR expression levels reported in different series have ranged from 25% to 90%, and EGFR expression has been shown to be associated with advanced stage, metastatic disease, and poor differentiation and survival [6,7,13,15,16,17]. Besides, EGFR and its downstream signaling effectors have vital roles in the development and progression of PDAC [16,18,19]. However, the significance of EGFR expression in terms of clinical outcomes is not well defined.

The current study investigated the association between EGFR expression and pancreatic cancer outcome in an East Asian population. We retrospectively examined the EGFR expression profiles of 357 resected PDAC specimens and analyzed the association of EGFR expression with prognosis in the overall population as well as the subgroups.

## 2. Results

### 2.1. Patient Characteristics According to EGFR (Epidermal Growth Factor Receptor) Expression

**Figure 1:**
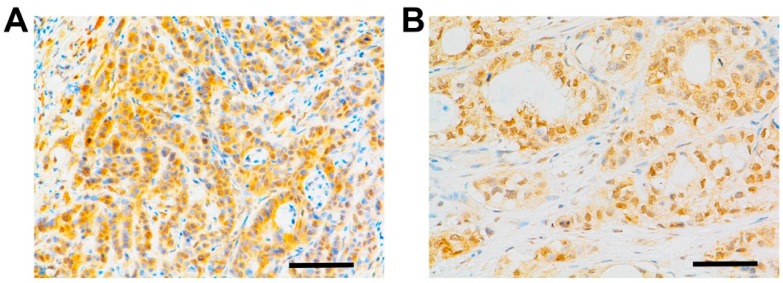

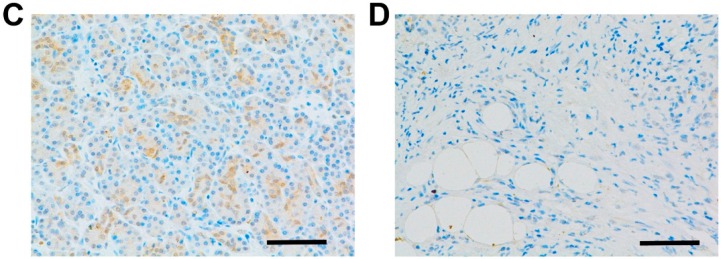
Immunohistochemical analysis of EGFR (epidermal growth factor receptor) in PDAC (pancreatic duct adenocarcinoma)tissues. In PDAC tissues, immunoreactivity for EGFR was observed on the surface and in the cytoplasm of cancer cells (**A**–**C**), with no immunoreactivity in the surrounding stroma (**D**). The immunoreactivity was different in respective cases: (**A**) strong; (**B**) moderate; (**C**) weak expression; and (**D**) absent (scale bars, 200 μm).

The clinicopathologic characteristics of 357 patients pathologically confirmed with PDAC were reviewed. EGFR expression was determined immunohistochemically on the surface and in the cytoplasm of cancer cells within resected tissues, but none was observed in the surrounding stroma (Figure 1). Membranous or cytoplasmic EGFR expression was detected in 54.6% (195 of 357) of the examined tissue specimens. One hundred three specimens (44.6%) of the 231 younger patients (age ≤ 65 years) and 59 specimens (45.2%) of the 126 elder patients (age > 65 years) were identified as EGFR-positive. The positive rates of EGFR expression were 48.3% (99/205) for females and 41.5% (63 of 152) for males. More detailed information of the patients’ characteristics and EGFR expression profiles are shown in Table 1. EGFR expression was not significantly associated with tumor size or location (*p* = 0.937 and 0.705, respectively) or CA19-9 level (*p* = 0.435). Furthermore, EGFR expression was not significantly correlated with metastasis to nerves, blood vessels and lymph nodes (*p* = 0.180, 0.931 and 0.736, respectively) (Table 1). EGFR expression in the resected tissues was not correlated with tumor proliferation (*p* = 0.154 for Ki67 analysis) or tumor stages (*p* = 0.473); however, there was a correlation with differentiation (*p* = 0.015) (Table 1).

### 2.2. EGFR Status Was Not Correlated with Overall Survival after Surgical Resection

The median survival of the overall population was 13.9 months. The associations of the clinical outcomes with demographic and prognostic factors were also investigated. Earlier tumor stages, non-lymph metastasis and EGFR negative expression were significantly related to a longer progression free survival (PFS) (*p* = 0.006, *p* = 0.001 and *p* = 0.040) (Table 1). The univariate analysis of median overall survival (OS) revealed that those patients: (a) with a lower CA19-9 level (≤37 U/mL), with a smaller tumor size (≤3 cm), with poorer differentiation and earlier stage; (b) with no lymph node or vascular invasion; and (c) treated with chemotherapy or radiotherapy showed a significantly longer survival than those with a different status (each *p*-value was <0.05) (Table 2). The remaining parameters were negatively associated with prognosis. Although EGFR expression was significantly associated with median PFS (*p* = 0.040), patients negative for EGFR expression were less likely to have a favorable prognosis than those positive for EGFR expression (median OS: 15.0 vs. 13.1 months, Hazard ratios (HR) = 1.07, *p* = 0.574), and this result is different from those of previous similar studies [6,16,17].

### 2.3. EGFR Status Was Not Correlated with Overall Survival after Surgical Resection Patients

CA19-9 level, tumor size, differentiation, stage, and vessel or lymph invasion, the potential prognostic factors, were significantly correlated with median OS (Table 2); however, no correlation was observed between EGFR expression and OS (Table 2 and Figure 1). The associations of each prognostic parameter with survival were compared between the EGFR-negative and EGFR-positive subgroups. The significant association between CA19-9 level and favorable prognosis, as observed in the overall population, was only detectable in the EGFR-negative subgroup (HR > 1.0, *p* < 0.050 for both univariate analysis and multivariate analysis); this result was also found in the associations of negative nerve invasion and negative vessel invasion with favorable prognosis. In contrast, tumor size was the only factor significantly correlated with OS in the EGFR-positive subgroup (HR > 2.0, *p* < 0.001 for both univariate analysis and multivariate analysis) (Table 3), and larger tumor size clearly had the highest HR (3.27) of all the prognostic factors evaluated. Lymph node metastasis had strong prognostic implications in both the EGFR-negative and EGFR-positive subgroups (HR > 1.0, *p* < 0.050 for both univariate analysis and multivariate analysis).

Furthermore, we investigated the prognostic value of postoperative adjuvant therapy in both EGFR subgroups. Chemotherapy was the only significant factor significantly associated with survival in the EGFR-positive subgroup, and the significance levels obtained in the univariate and multivariate analyses were comparable; however, radiotherapy demonstrated only a slight advantage in the EGFR-positive subgroup using the univariate analysis. In contrast to chemotherapy, radiotherapy showed a significant survival benefit in the EGFR-negative subgroup in both the univariate and multivariate analyses, and only a slight advantage was found in the univariate analysis (*p =* 0.055) in the EGFR-positive subgroup. Likewise, age, gender and tumor location were parameters all weakly associated with survival (Table 3). Additionally, stratified by EGFR expression, patients with smaller size, non-lymph metastasis and accepted radiotherapy showed longer median PFS in the EGFR positive group (*p* < 0.05); however, patients with non-nerve invasion were found to have a better PFS in the EGFR negative group (*p =* 0.022) (Supplementary Table S1).

### 2.4. EGFR Status Was Not Correlated with Overall Survival after Surgical Resection Patients

**Figure 2:**
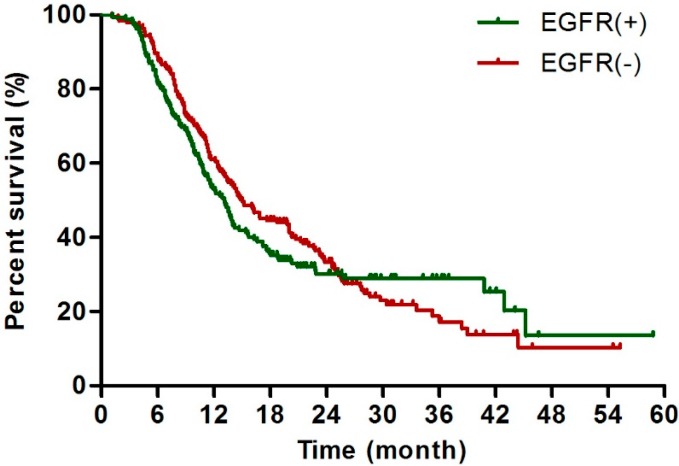
Comparison of overall survival between each EGFR status. Kaplan–Meier Estimates of survival according to EGFR expression (EGFR (+) or EGFR (−)).

The Kaplan–Meier curves revealed a similar prognostic value of EGFR expression in terms of short-term survival. The OS curves stratified by positive and negative EGFR expression were shown in Figure 2 and suggested that EGFR expression in resected pancreatic cancer was not correlated with survival; this result was in accordance with the statistical analysis findings presented in Table 2. The Kaplan–Meier curve also indicated that patients with negative EGFR expression had a longer median PFS than others with positive EGFR expression within approximately 30 months (Supplementary Figure S1). The statistical analysis showed a negative correlation between chemotherapy treatment and EGFR expression (Table 1). However, undergoing chemotherapy or radiotherapy was independently and highly significantly correlated with favorable outcomes (Table 2).

**Figure 3:**
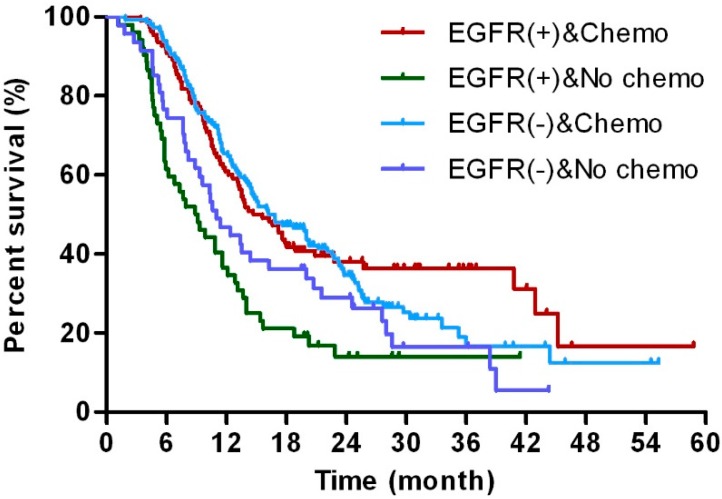
Subgroup analysis of adjuvant chemotherapy. Kaplan–Meier estimates of survival of patients who received adjuvant chemotherapy (Chemo) or refused chemotherapy (No chemo) according to EGFR status: EGFR (+) or EGFR (−).

**Figure 4:**
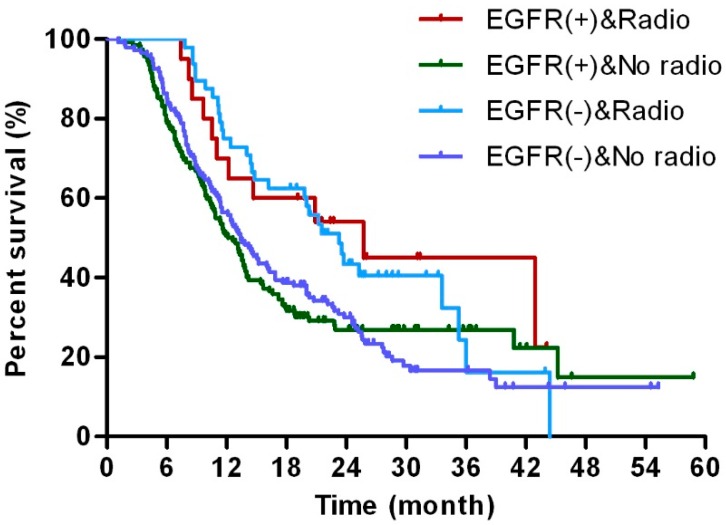
Subgroup analysis of adjuvant radiotherapy. Kaplan–Meier estimates of survival of patients who received adjuvant radiotherapy (Radio) or refused radiotherapy (No radio) according to EGFR status: EGFR (+) or EGFR (−).

The prognosis of patients treated with adjuvant therapy was analyzed in both the EGFR-positive (EGFR (+)) and EGFR-negative (EGFR (−)) subgroups (Table 3). To further determine the correlation between postoperative chemotherapy (chemo) and EGFR expression, all patients with available final survival data were divided into four Groups: EGFR (+) and chemo; EGFR (+) and no chemo; EGFR (−) and chemo; EGFR (−) and no chemo. The investigation of OS in these Groups showed a benefit of postoperative chemotherapy in both the EGFR (+) and EGFR (−) subgroups. Particularly, EGFR-positive patients who underwent chemotherapy showed increased survival compared to those who without chemotherapy; however, this difference was less obvious in the EGFR-negative subgroup (Figure 3). Similarly, patients who received radiotherapy following radical resection showed increased survival compared with those without, but this difference was not statistically insignificant in the EGFR (+) and EGFR (−) subgroups (Figure 4). However, postoperative chemotherapy was effective in preventing the tumor progression among the EGFR negative patients, which was not detected in the EGFR positive cases. Moreover, this effect was not detected in the postoperative radiotherapy (Supplementary Figure S2).

## 3. Discussion

Although radical resection can be curative, the median OS of PDAC patients following radical resection is less than 20 months. Early metastasis and recurrence are the main reasons for the dismal prognosis of this disease even after curative resection. Distinguishing patients who are at high risk recurrence and distant organ metastasis after surgery is crucial for an improved survival since conventional factors are not completely conclusive and potent. Therefore, the prognostic ability of EGFR in pancreatic cancer is an ongoing topic of discussion.

We conducted a retrospective study of EGFR expression in postoperative patients and investigated its correlation with the clinical outcomes of PDAC patients. To the best of our knowledge, this study is the largest population-based study to evaluate EGFR expression as a potential prognostic marker in East Asian patients with resectable PDAC. Consistent with some recent studies, we showed that the rate of EGFR expression was 45.4% among the pancreatic cancer patients, as tested by a total of 357 patients with pancreatic carcinoma [20]. We revealed that EGFR expression’s slight correlations with age, gender, CA19-9, and tumor site, size, stage, and grade. Additionally, the EGFR expression can serve as an independent marker indicative of tumor invasion and metastasis Radiotherapy was found to be a confounder of the distribution of EGFR express, and this result should be verified by future studies.

Previous reports showed that EGFR over-expression may predict shorter survival in multiple solid tumors, including pancreatic tumors [6,14,17]. A prior study found a significant correlation between high EGFR expression and shorter overall survival (*p* = 0.014) in 88 surgical patients by measuring mRNA levels in paraffin-embedded (FFPE) tissues [17]. In addition, the expression of EGFR was reported in conjunction with insulinlike growth factor 1 receptor (IGF-1R) to predict poor survival in pancreatic ductal adenocarcinoma [21]. However, we were unable to detect a significant difference in OS between the EGFR-positive and EGFR-negative subgroups (OS: 15.0 vs. 13.1 months, *p* = 0.98). Similar studies also reported the deficiency of correlation between EGFR expression and overall survival [22,23]. These conflicting results may be partially due to the differences in the patient populations between the previous study and our study, including ethnic differences, various ratios of resectable to advanced patients, and inclusion of multiple types of pancreatic cancer. Additionally, differences in the laboratory methodologies used when analyzing overall survival could also have contributed to the conflicting results, and these findings are consistent with a previous systematic meta-analysis [24].

The high rate of recurrence observed indicated the enormous difficulties to eliminate microscopic systemic spread by surgical resection alone; thus, adjuvant therapies must be investigated to improve postoperative survival in PDAC patients. Gemcitabine-based and 5-FU-based adjuvant chemotherapies have been widely accepted to be capable of increasing OS in patients with resected pancreatic cancer; however, the optimization between therapeutic regimens is controversial [4,8,25]. The high rate of recurrence suggests that adjuvant therapies are necessary for even R0 resected disease to improve survival outcomes in these patients. Although we repeatedly stressed the significance of adjuvant therapy, 99 patients had persisted to refuse any adjuvant therapy. The survival analysis of this study showed that both adjuvant chemotherapy and radiotherapy following resection have significant survival benefits in PDAC patients (Table 2). Additionally, our results suggest that EGFR-positive patients are more likely to benefit from adjuvant chemotherapy than EGFR-negative patients, indicating greater sensitivity to chemotherapy in EGFR-positive patients. Conversely, adjuvant radiotherapy only improved survival in the EGFR-negative subgroup, and this finding requires confirmation by prospective randomized clinical trials. The approaches to enhance sensitivity to chemotherapy and radiotherapy are complex, and particularly relevant for signaling pathways involved in metabolism and apoptosis [9,26]. As far as we know, there is no precise mechanism, related to EGFR expression regulating the chemotherapy effects, has been reported in pancreatic cancer. According to a recent study, Kim et al. found that EGFR expression had better overall survival in the gastric cancer patients who received adjuvant chemotherapy [27]. Additional evidences has also been suggesting that cytotoxic chemotherapy is more effective among patients with high EGFR expression than in those with low EGFR expression in non-small-cell lung cancer (NSCLC) and colorectal cancer (CRC), respectively [28,29]. The prognostic and predictive roles of EGFR expression in pancreatic cancer remain controversial.

In pancreatic cancer, overexpressed EGFR regulates downstream signaling activation, including PKC, PI3K/AKT/mTOR, SRC, STAT and RAS/RAF/MEK1/ERK1/2, and regulate numerous EGFR-interacting proteins [30,31]. Recently, one study reported that dysregulation of ubiquitination is a key mechanism of EGFR hyperactivation in PDAC, and low CBL (Casitas B-lineage lymphoma family) may define PDAC tumors likely to respond to erlotinib treatment [32]. Another study identified the lumican/EGFR/AKT/HIF1 α signaling pathway as a mechanism to inhibit pancreatic cancer cell survival and proliferation and prolonged survival after tumor resection, which stressed that lumican plays a restrictive role in EGFR-expressing pancreatic cancer progression [18]. Based on those previous studies, EGFR and the related pathway had shown an important role in pancreatic cancer. Therefore, it is rational to label EGFR as a novel target for treatments of pancreatic cancer.

Adjuvant therapy had shown the survival benefit, but the median OS for patients with resected pancreatic cancer still remains less than 20 months. Multiple trials have been performed to explore other systemic therapies in the adjuvant setting [33,34,35]. In a wide range of human solid carcinomas, molecules involved in EGFR signaling that have aberrant expression were reported as new therapeutic targets [14]. Various therapeutic agents designed to target EGFR and EGFR-associated pathways are currently being developed, including specific antibodies, flavonoid antioxidants, and small molecular EGFR-specific tyrosine kinase inhibitors [5,36]. Preclinical studies of those agents in pancreatic carcinoma have had positive results [37,38,39]. Although gemcitabine is a key adjuvant chemotherapy agent for advanced pancreatic cancer, the combination of agents targeting EGFR and gemcitabine has been shown to be superior to gemcitabine alone [39,40]. Therefore, we hypothesized that EGFR-targeted adjuvant therapy combined with gemcitabine may be a promising therapeutic option in resectable pancreatic cancer patients with positive EGFR expression. It is believed that future clinical trials of EGFR inhibitors should incorporate systematic evaluations of both cytoplasmic and membrane EGFR expression, especially in adjuvant settings.

## 4. Materials and Methods

### 4.1. Patients

We retrospectively evaluated PDAC patients who had been treated at Fudan University Shanghai Cancer Center from 2007 to 2014. A total of 357 patients, 205 males (57%) and 152 females (43%), were histologically diagnosed with ductal adenocarcinoma and underwent surgical R0 resection. R0 resection was defined as absence of macroscopic and microscopic residual tumor >1 mm from the margin of the resection specimen. Patients with pancreatic endocrine or acinar pancreatic carcinoma were excluded from this study. Written informed consent was obtained from all patients before they participated in the study. The study was conducted in accordance with the Declaration of Helsinki, and the protocol was approved by the Ethics Committee of Fudan University Shanghai Cancer Center.

### 4.2. Adjuvant Therapy

In this work, 258 patients accepted chemotherapy and 99 cases did not, and none of the 99 patients accepted any adjuvant therapy including radiotherapy, reasons for which included bad performance status or postoperative complications. The adjuvant chemotherapy regimens included gemcitabine-based (94.4%) and fluoropyrimidine-based (5-FU-based, 5.6%) protocols. In the patients who underwent radiotherapy, the primary tumors and surrounding regions that were thought to affect overall survival were targeted. The study was approved by the ethics committee of Fudan University Shanghai Cancer Center. Written informed consent was obtained from all patients, and the clinical factors were evaluated based on the original histopathology reports and clinical records.

### 4.3. Tissue Samples

Patients with well-defined PDAC were eligible for inclusion if an excised tumor specimen was available for examination of EGFR expression. Ten percent neutral-buffered, formalin-fixed, paraffin-embedded tissue blocks were obtained from surgical resection specimens and cut into 4-μm sections. For pathologic diagnosis, the tissue sections were stained using hematoxylin-eosin. Most of these procedures were performed by the Department of Pathology, Fudan University Shanghai Cancer Center.

### 4.4. Immunohistochemistry

Immunohistochemical analysis of the formalin-fixed, paraffin-embedded (FFPE) tumor tissues for EGFR expression was performed using the Dako EGFR PharmDx TM kit (Dako, Glostrup, Denmark). The FFPE specimens were evaluated for both cytoplasmic and membranous immunostaining by two independent pathologists blinded to the clinical results (recorded at Fudan University Shanghai Cancer Center). The cases were divided into EGFR-positive and EGFR-negative subgroups based on the intensity and completeness of immunohistochemical staining. The staining intensity was scored as 0 for absent, 1 for weak, 2 for moderate and 3 for strong staining. The staining distribution was scored as 0 (absent) for <5%, 1 (focal) for 5% to <50%, and 2 (diffuse) for ≥50% positive-stained areas. The sum of intensity and distribution scores were then used to determine the EGFR immunoreactivity. For membranous or cytoplasmic staining, the intensity score of 1 and 0, or the distribution score of 0, was considered as negative immunoreactivity. Other scores were considered positive immunoreactivity.

### 4.5. Statistical Analysis

The statistical analysis was performed using SPSS 17.0 statistical software (Chicago, IL, USA). All *p*-values <0.050 were considered statistically significant. Survival time was defined as the time from radical operation completion to the last contact with the patient or death from the disease. Progression free survival was defined as the time from surgery performed to the first recurrence detected. All the subjects were followed for at least 18 months or until death from the disease. Kaplan–Meier analysis was used to analyze patient survival and calculate survival curves, and the Mantel–Cox log-rank test was used to determine the differences between the curves.

## 5. Conclusions

In summary, although EGFR status was not able to predict postoperative prognosis based on our data, EGFR expression is strongly correlated with adjuvant treatment outcome. Additional studies are necessary to elucidate the potential mechanisms of the effects of EGFR on tumorigenesis and the significance of EGFR in the treatment of human pancreatic cancer.

## Figures and Tables

**Table 1:** Characteristics of patients with pancreatic cancer according to EGFR (epidermal growth factor receptor) expression.

EGFR	Total	Positive vs. Negative		
		Negative	Positive	*p*-Value
Age, years				
≤65	231	128	103	0.685
>65	126	67	59	
Gender				
Male	205	106	99	0.199
Female	152	89	63	
Tumor location				
Head	211	117	94	0.705
Others	146	78	68	
CA 19-9 (U/mL)				
≤37	84	49	35	0.435
>37	273	146	127	
Size (cm)	3.5 ± 1.6	3.5 ± 1.7	3.5 ± 1.5	0.937
Differentiation				
well	7	6	1	0.015
moderate	200	119	81	
poor	136	61	75	
unknown	14	9	5	
Tumor stages				
IA	24	10	14	0.473
IB	91	54	37	
IIA	89	49	40	
IIB	153	82	71	
Nerve invasion				
yes	301	169	132	0.180
no	56	26	30	
Vessel invasion				
yes	72	39	33	0.931
no	285	156	129	
Lymph metastasis				
yes	153	82	71	0.736
no	204	113	91	
Chemotherapy				
yes	258	148	110	0.093
no	99	47	52	
Radiotherapy				
yes	68	48	20	0.003
no	289	147	142	
Ki67 (%)	34.0 ± 21.6	32.4 ± 20.9	35.8 ± 22.3	0.154

Adjuvant chemotherapy was not associated with EGFR expression (*p* = 0.093), but EGFR expression was significantly correlated with the receipt of radiotherapy (*p* = 0.003).

**Table 2:** Hazard ratios (HR) and *p*-value for overall survival (OS) and progression free survival (PFS) associated with conventional prognostic factors and EGFR expression.

Variables (*n*)	No.	Median PFS (Months)	*p*-Value	Median OS (Months)	Univariate		Multivariate	
					HR	*p*-Value	HR	*p*-Value
Age, years								
≤65	231	10.5	0.429	14.7	1	0.082	-	-
>65	126	11.4		13.2	1.25		-	
Gender								
Male	205	10.5	0.205	13.8	1	0.204	-	-
Female	152	11.8		14.2	0.85		-	
Tumor location								
Head	211	10.8	0.696	14.0	1	0.402	-	-
Others	146	11.2		13.5	0.90		-	
CA19-9 (U/mL)								
≤37	84	12.3	0.202	17.6	1	0.039	1	0.026
>37	273	10.7		13.2	1.35		1.42	
Size (cm)								
≤3	178	11.8	0.172	17.6	1	0.001	1	0.000
>3	179	10.3		11.0	1.61		1.67	
Differentiation								
poor	136	10.4	0.209	14.5	1	0.015	1	0.000
moderate	200	11.1		17.7	0.705		0.716	
well	7	19.0		30.6	0.346		0.385	
unknown	14	12.8		17.7	0.776		0.781	
Tumor stages								
IA	24	14.4	0.006	21.0	1	0.000	1	0.000
IB	91	12.1		19.0	1.56		1.39	
IIA	89	12.1		17.8	1.73		1.46	
IIB	153	9.27		14.0	2.66		2.45	
Nerve invasion								
yes	301	10.7	0.144	13.8	1	0.338	-	-
no	56	12.9		14.2	0.85		-	
Vessel invasion								
yes	72	9.1	0.092	9.3	1	0.002	1	0.076
no	285	11.3		15.0	0.63		0.75	
Lymph metastasis								
no	153	9.3	0.001	16.9	1	0.000	1	0.000
yes	204	12.4		10.9	1.71		1.78	
Chemotherapy								
yes	258	11.6	0.113	16.1	1	0.000	1	0.009
no	99	9.7		10.3	1.77		1.46	
Radiotherapy								
yes	68	13.1	0.062	23.3	1	0.001	1	0.006
no	289	10.6		13.1	1.71		1.70	
EGFR								
Negative	195	12.2	0.040	15.0	1	0.574	1	0.986
Positive	162	9.6		13.1	1.07		1.00	

According to the multivariate analysis, CA19-9, tumor size, lymph node metastasis, and receipt of chemotherapy or radiotherapy were significantly associated with overall survival (detailed information is shown in Table 2). Negative vessel invasion was strongly correlated with a favorable outcome in the univariate analysis (HR = 0.63, *p* = 0.002), but this correlation had borderline significance in the multivariate analysis (HR = 0.75, *p* = 0.076).

**Table 3:** Hazard ratios (HR) and *p*-value for death associated with demographic and prognostic factors stratified by EGFR expression using univariate and multivariate analyses.

Characteristics	EGFR (+)				EGFR (−)			
	Univariate Analysis		Multivariate Analysis		Univariate Analysis		Multivariate Analysis	
	HR	*p*-Value	HR	*p*-Value	HR	*p*-Value	HR	*p*-Value
Age > 65	1.30	0.176	-	-	1.19	0.318	-	-
Female	0.96	0.820	-	-	0.79	0.165	-	-
Head	1.10	0.605	-	-	1.11	0.542	-	-
CA19-9 > 37 U/mL	1.22	0.381	-	-	1.55	0.030	1.64	0.016
Size > 3 cm	2.36	0.000	2.38	0.000	1.20	0.272	-	-
Nerve invasion	0.69	0.112	-	-	2.03	0.012	1.71	0.063
Vessel invasion	1.28	0.273	-	-	1.99	0.000	1.67	0.015
Lymph metastasis	1.79	0.002	1.97	0.000	1.67	0.002	1.59	0.006
Chemotherapy	0.47	0.000	0.54	0.002	0.70	0.061	0.80	0.280
Radiotherapy	0.54	0.055	0.65	0.201	0.60	0.013	0.63	0.029

## Supplementary Materials

Supplementary materials can be found at http://www.mdpi.com/1422-0067/17/7/1090/s1.
