# Supplementary material for: The Prognostic and Predictive Role of Epidermal Growth Factor Receptor in Surgical Resected Pancreatic Cancer
Source: Int J Mol Sci. 2016 Jul 8;17(7):1090. doi: 10.3390/ijms17071090 (PMC4964466; doi:10.3390/ijms17071090)
Supplement: Supplementary file 1 [file ijms-17-01090-s001.pdf]

Supplementary Materials: The Prognostic and Predictive Role of Epidermal Growth Factor Receptor in Surgical Resected Pancreatic Cancer

Meng Guo, Guopei Luo, Chen Liu, He Cheng, Yu Lu, Kaizhou Jin, Zuqiang Liu, Jiang Long, Liang Liu, Jin Xu, Dan Huang, Quanxing Ni and Xianjun Yu

Table S1. Hazard ratios (HR) and *p* value for progress free survival (PFS) associated with prognostic factors stratified by EGFR expression using multivariate analyses.

Characteristics	Multivariate Analysis of PFS by EGFR (+)		Multivariate Analysis of PFS by EGFR (–)	
	HR	*p*-Value	HR	*p*-Value
Age > 65	1.00	0.973	1.00	0.885
Female	0.73	0.059	1.01	0.941
Head	0.99	0.936	0.92	0.577
CA19-9 > 37 U/mL	0.69	0.059	0.881	0.469
Size ≤ 3 cm	0.69	0.037 *	1.12	0.473
Well differentiation	0.17	0.082	0.73	0.509
Advanced stage	1.31	0.391	0.54	0.073
Nerve invasion	1.11	0.621	0.58	0.022 *
Vessel invasion	1.02	0.923	0.92	0.645
Lymph metastasis	1.48	0.023 *	1.22	0.195
Chemotherapy	1.20	0.308	1.137	0.489
Radiotherapy	2.08	0.003 *	0.852	0.377

* *p*-Value less than 0.05.

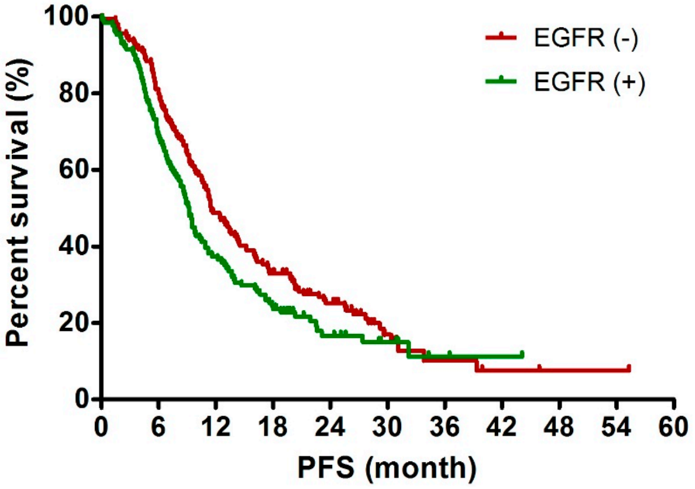

Figure S1. Comparison of PFS between each EGFR status. Kaplan–Meier test showed that PDAC patients with negative EGFR expression (EGFR (−)) had a longer median PFS than others with positive EGFR expression (EGFR (+)) within a medium-short term (approximate 30 months).

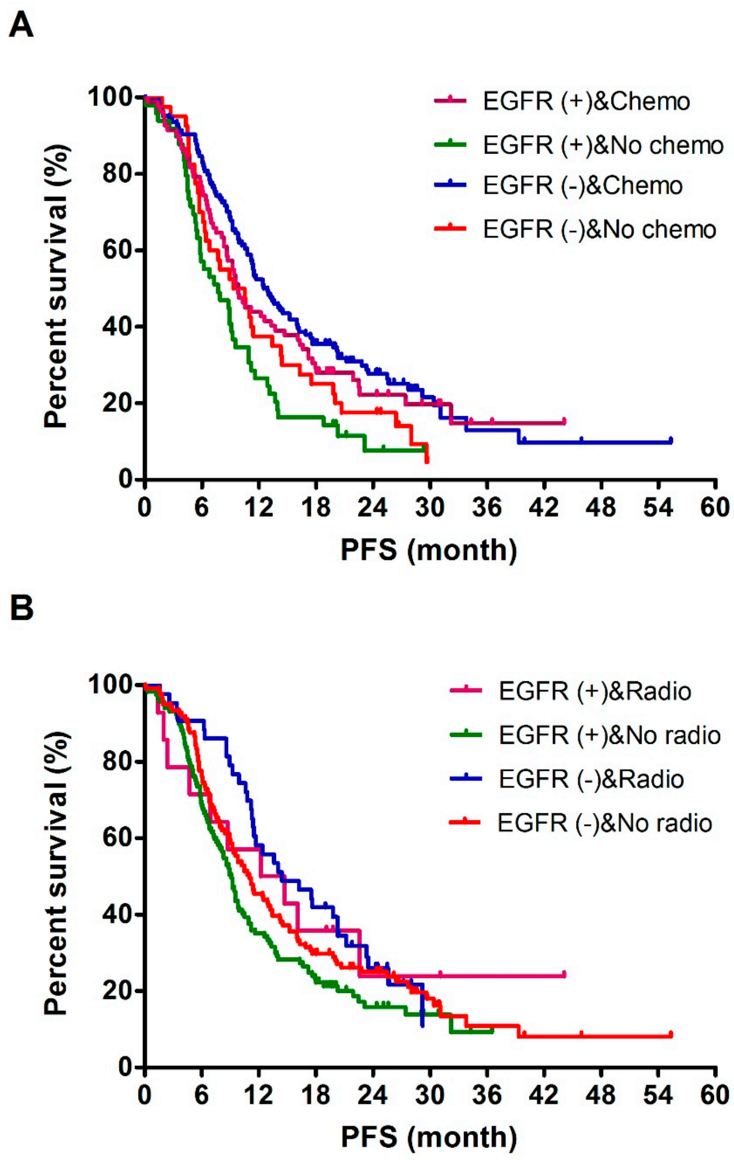

Figure S2. Stratified analysis of the relationship between PFS and adjuvant chemotherapy by EGFR status. Postoperative chemotherapy was effective to prolong the PFS among the EGFR negative patients, which similar effect was also detected between the EGFR positive groups (**A**); Treated with postoperative radiotherapy, the PFS was prolonged among the EGFR positive patients, however, not prolonged among the EGFR negative cases (**B**).
